# TINCR is not a non‐coding RNA but encodes a protein component of cornified epidermal keratinocytes

**DOI:** 10.1111/exd.14083

**Published:** 2020-02-18

**Authors:** Leopold Eckhart, Julia Lachner, Erwin Tschachler, Robert H. Rice

**Affiliations:** ^1^ Department of Dermatology Medical University of Vienna Vienna Austria; ^2^ Department of Environmental Toxicology University of California Davis CA USA

**Keywords:** cornification, differentiation, epidermis, evolution, ubiquitin

## Abstract

Long non‐coding RNAs have been implicated in the regulation of a plethora of biological processes, yet it has been challenging to verify that they are truly not coding for proteins. Terminal differentiation‐induced non‐coding RNA (TINCR) is a 3.7‐kilobase mRNA that is highly abundant in epidermal keratinocytes prior to cornification. Here, we report the presence of an evolutionarily conserved open reading frame in *TINCR* and the identification of peptides derived from this open reading frame in the proteome of human stratum corneum. Our results demonstrate that TINCR is a protein‐coding RNA and suggest that the TINCR‐encoded protein is involved in keratinocyte cornification.

## 1 BACKGROUND

Long non‐coding RNAs (lncRNAs) are RNAs of at least 200 nucleotides length that are not translated into proteins. They represent a heterogeneous group of RNAs including mRNA‐like intergenic transcripts (lincRNAs), antisense transcripts of protein‐coding genes and others.^1^ The functions of many lncRNAs are not known but some lncRNAs were shown to control nuclear architecture and transcription in the nucleus and to modulate mRNA stability and translation in the cytoplasm.^1^ lncRNAs as potential regulators of many cellular processes have sparked great interest among researchers in dermatology, and several important roles of lncRNAs in skin cells have been demonstrated.^2^, ^3^, ^4^, ^5^, ^6^, ^7^

Terminal differentiation‐induced non‐coding RNA (TINCR) was identified in differentiating epidermal keratinocytes.^8^ *TINCR* RNA contains so‐called “TINCR box” motifs, which are 25 nucleotides long and were reported to mediate the interaction with “TINCR box” motifs in multiple cellular mRNAs. Furthermore, *TINCR* RNA reportedly binds to the staufen1 protein and subsequently stabilizes keratinocyte differentiation‐associated mRNAs.^8^ Depletion of TINCR and staufen1 impaired differentiation of keratinocytes, suggesting that TINCR is essential for this process. Subsequent studies revealed transcription factor signalling through MAF:MAFB as downstream targets of TINCR.^9^ Additional mechanisms of action and various mechanism of regulation of TINCR in skin and other organs have been reported in recent years.^4^, ^10^, ^11^

TINCR was first cloned from human hippocampus in the course of the National Institutes of Health, Mammalian Gene Collection project and was originally designated “Homo sapiens placenta‐specific 2 (non‐protein coding), mRNA” (GenBank accession number BC036545).^12^ Alternative names such as LINC00036, NCRNA00036 and onco‐lncRNA‐16 supported the non‐coding nature of this RNA. Automated analysis of the DNA sequence of human chromosome 19^13^ led to the identification of an open reading frame in *TINCR* that was deposited in the Uniprot database under the accession number A0A1B0GVN0. The Uniprot database was used as a reference for the proteomic analysis of human stratum corneum, and peptides corresponding to TINCR were identified in cornified envelopes.^14^

## 2 QUESTIONS ADDRESSED

Here, we address the question as to whether TINCR is a non‐coding or a protein‐coding RNA.

## 3 EXPERIMENTAL DESIGN

We obtained amino acid sequences of peptides from a mass spectrometry (MS) analysis of human stratum corneum proteins that was reported in detail previously.^14^ In brief, cornified envelopes were collected with adhesive discs from healthy forearm skin, eluted, incubated with SDS‐dithioerythritol and separated into a solubilized and an insoluble (envelope) fraction which were analysed by liquid chromatography‐MS/MS.^14^, ^15^ RNA was prepared from the skin of chickens and subjected to reverse‐transcription polymerase chain reaction (RT‐PCR) with the intron‐spanning primers GgTINCRs 5′‐GGATGCTCCTCTCTGCCACA‐3′ and GgTINCRa 5′‐CACGCTGCGTTCCATGGTCA‐3′. The PCR product was sequenced (GenBank accession number MN857541). The open reading frames of human TINCR and TINCR of other species were translated, and the resulting amino acid sequences were aligned with the Multalin algorithm.^16^ Amino acid sequences were subjected to Conserved Domain search at https://www.ncbi.nlm.nih.gov/Structure/cdd/wrpsb.cgi.^17^

## 4 RESULTS

**Figure 1:**
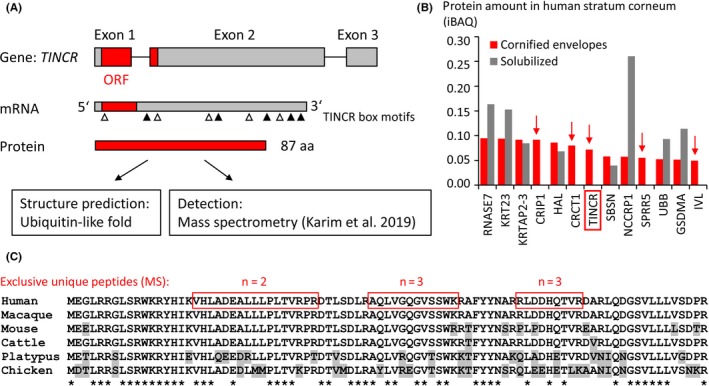
TINCR encodes an evolutionarily conserved protein. A, Schematic depiction of the protein‐coding role of TINCR. Exons are depicted as boxes with the open reading frame (ORF) shown in red. The coding region and the relative positions of so‐called “TINCR box” sequence motifs (white and black triangles) are indicated on the mRNA. B, Protein amounts in cornified envelopes and in the solubilized fraction of human stratum corneum are shown in units of intensity‐based absolute quantification (iBAQ). The values were obtained from Tables S2 and S3 of Reference Karim et al 2019.^14^ Proteins detected only in the cornified envelope fraction are marked by red arrows. C, Amino acid sequence alignment of TINCR proteins from phylogenetically diverse mammals and chicken. Exclusive unique peptides detected by mass spectrometry (MS)^14^ are indicated by red boxes in the human TINCR sequence. Numbers (n) of peptide hits are shown above the boxes. Grey shading indicates amino acid residues different from the human counterpart. Residues identical in all TINCR orthologs are indicated by asterisks below the alignment

Human *TINCR* RNA is produced by transcription of the *TINCR* gene which is located at chromosome 19p13.3 and comprises 3 exons (Figure 1A). Peptides identified in stratum corneum^14^ correspond to a protein encoded by an open reading frame (ORF) that spans two exons of *TINCR* (Figure 1A, Figure S1). The corresponding translated protein has a length of 87 amino acid residues and is predicted to fold into a ubiquitin‐like 3‐dimensional structure (Figure S2).

Semi‐quantitative analysis of stratum corneum proteins suggests that the abundance of TINCR is in a similar range as that of established keratinocyte differentiation proteins such as Rnase7, histidase (HAL) and involucrin (IVL) (Figure 1B). Like two cysteine‐rich cornification proteins (CRIP1 and CRCT1) and two substrates of cornification (SPRR5 and IVL), TINCR was detected in the cross‐linked fraction of the cornified envelope protein but not in the solubilized protein fraction (Figure 1B),^14^ suggesting that TINCR is efficiently integrated into cornified envelopes.

Amino acid sequence analysis showed that TINCR does not contain cysteine residues, whereas glutamine and lysine residues are present as potential sites of transglutamination (Figure 1C). Comparison of amino acid sequences of TINCR orthologs showed high degrees of sequence conservation among mammals and more than 50% sequence identity to a predicted TINCR protein of the chicken (Figure 1C; Figure S1), suggesting that the open reading frame of *TINCR* has been conserved since the evolutionary divergence of the lineages leading to mammals and birds more than 300 million years ago.^18^

## 5 CONCLUSIONS

The presence of TINCR peptides in human stratum corneum and the conservation of the TINCR open reading frame through evolution indicate that TINCR encodes a protein. It is intriguing that TINCR is predicted to fold into a ubiquitin‐like domain which may facilitate specific interactions with other proteins. TINCR RNA is expressed in the skin where it is confined to differentiating keratinocytes of epidermis^8^ and at lower levels in the oesophagus and placenta. Proteins of these tissues should be investigated for binding partners of TINCR protein. Importantly, as deduced from the mass spectroscopic analysis TINCR protein persists throughout cornification of keratinocytes so that it is detectable in the stratum corneum.^14^ Whether TINCR acts primarily as component of cornified envelopes or whether it has other functions during cornficiation remains to be determined in future studies.

Previously, TINCR was classified as a long non‐coding RNA and interactions of TINCR RNA with protein‐coding mRNAs, miRNAs and proteins were suggested to mediate effects of TINCR on keratinocyte differentiation. These interactions were not investigated in the present study, and therefore, binding of TINCR RNA to other molecules is not excluded. However, the fact that *TINCR* encodes a protein is definitely not compatible with its current designation as non‐coding RNA. Thus, we propose that TINCR should stand for Terminal differentiation‐INduced Cornification Regulator.

## CONFLICT OF INTEREST

The authors have no conflict of interest.
