# Supplementary material for: TINCR is not a non‐coding RNA but encodes a protein component of cornified epidermal keratinocytes
Source: Exp Dermatol. 2020 Feb 18;29(4):376–9. doi: 10.1111/exd.14083 (PMC7187231; doi:10.1111/exd.14083)
Supplement: Supplementary file 1 [file EXD-29-376-s001.pdf]

```
                                M  E  G  L  R  R  G  L  S  R  W  K  R  Y  H  I  K  V
Human     AGCCGGAGCCGGGCGGGCGCCATGGAGGGGCTGCGGCGGGGGCTGTCGCGCTGGAAGCGCTACCACATCAAGGTG
Chicken   AGCCGGAGAGG----------ATGGACACACTGCGAAGAAGCCTTTCTCGCTGGAAGAGGTACCACATTAAGGTG
                                M  D  T  L  R  R  S  L  S  R  W  K  R  Y  H  I  K  V

           H  L  A  D  E  A  L  L  L  P  L  T  V  R  P  R  D  T  L  S  D  L  R  A  Q
Human     CACCTGGCGGACGAGGCGCTGCTGCTACCGCTGACCGTGCGGCCGCGGGACACGCTCAGCGACCTGCGCGCCCAG
Chicken   CACTTGGCTGATGAGGACCTGATGATGCCGCTGACCGTCAAGCCCAGAGACACAGTGATGGACCTACGGGCTTAC
           H  L  A  D  E  D  L  M  M  P  L  T  V  K  P  R  D  T  V  M  D  L  R  A  Y

           L  V  G  Q  G  V  S  S  W  K  R  A  F  Y  Y  N  A  R  R  L  D  D  H  Q  T
Human     CTGGTGGGCCAGGGCGTGAGCTCCTGGAAGCGCGCCTTCTACTACAACGCGCGGCGGCTGGACGACCACCAGACG
Chicken   CTAGTACGGGAGGGCGTCACTTCCTGGAAGAAAACATTTTATTACAACTCCAGGCAGCTTGAAGAGCATGAGACT
           L  V  R  E  G  V  T  S  W  K  K  T  F  Y  Y  N  S  R  Q  L  E  E  H  E  T

           V  R  D  A  R  L  Q  D  G  S  V  L  L  L  V  S  D  P  R
Human     GTGCGCGACGCGCGCCTGCAGGACGGCTCGGTGCTGCTGCTCGTCAGCGACCCCAGGTGGCCGCGGNNNNNNNNN
Chicken   TTGAAAGCAGCCAATATCCAGAACGGCTCAGTCCTGCTTCTTGTCAGCAACAAAAGGTAGGCAAGGNNNNNNNNN
           L  K  A  A  N  I  Q  N  G  S  V  L  L  L  V  S  N  K  R

                       *
Human     NCCTCTTTCAGGTAGTCTGGGTTGGAGGAGGCAGAGCCATGACCAA-GGGGACCTGGGTACTGGCTGAAGGAATA
Chicken   NTCTTATGCAGATAACGCCAAAGAGCACAGCAAAAGCCATGGAGACGGGGAGCGTGACAGGAGCTGAAAGGAAG
                       *
```

Supplementary Figure S1. Nucleotide sequence alignment of human and chicken *TINCR* genes. Nucleotide sequences of the coding segments and their flanking regions within human and chicken *TINCR* were aligned with the Multalin algorithm. Coding sequence is indicated by yellow shading. Amino acid sequences of the encoded proteins are shown above and below the nucleotide seqeuences. An asterisk indicates the end of a protein. The sequence of the intron between the two coding exons is shaded grey. Only the first and the last 10 nucleotides of the intron are shown whereas the main portion of the intron is replaced by N's. Intronic splicing signals GT and AG are underlined. Red fonts indicate identical nucleotides both sequences. Dashes were introduced to optimize the alignment in the noncoding regions. GenBank accession numbers of the nucleotide sequences shown in the figure: Human TINCR (partial sequence), NC_000019.10, nucleotides 5567945-5567655 and 5562221-5562149 (reverse complement); Chicken TINCR (partial sequence), NC_006115.5, nucleotides 4549790-4550070 and 4556982-4557055. The expression of the protein-coding segment of chicken TINCR was confirmed by RT-PCR analysis of chicken skin with the primers GgTINCRs 5'-GGATGCTCCTCTCTGCCACA-3' and GgTINCRa 5'-CACGCTGCGTTCCATGGTCA-3' and sequencing of the PCR product (GenBank accession number MN857541).

List of domain hits

Name	Accession	Description	Interval	E-value
Ubl_Ddi1_like	cd01796	ubiquitin-like (Ubl) domain found in the eukaryotic Ddi1 family	16-83	4.76e-07

ubiquitin-like (Ubl) domain found in the eukaryotic Ddi1 family; The eukaryotic Ddi1 family, including yeast aspartyl protease DNA-damage inducible 1 (Ddi1) and Ddi1-like proteins from vertebrates and other eukaryotes, has been characterized by containing an N-terminal ubiquitin-like (Ubl) domain and a conserved retroviral aspartyl-protease-like domain (RVP) that is important in cell-cycle control. Yeast Ddi1 and many family members also contain a C-terminal ubiquitin-association (UBA) domain, however, Ddi1-like proteins from all vertebrates lack the UBA domain. Ddi1, also termed v-SNARE-master 1 (Vsm1), is an ubiquitin receptor involved in the cell cycle and late secretory pathway in Saccharomyces cerevisiae. It functions as an UBA-Ubl shuttle protein that is required for the proteasome to enable ubiquitin-dependent degradation of its ligands. For instance, Ddi1 plays an essential role in the final stages of proteasomal degradation of Ho endonuclease and of its cognate FBP, Ufo1. Moreover, Ddi1 and its associated protein Rad23p play a cooperative role as negative regulators in yeast PHO pathway. This family also includes mammalian regulatory solute carrier protein family 1 member 1 (RSC1A1), also termed transporter regulator RS1 (RS1), which mediates transcriptional and post-transcriptional regulation of Na(+)-D-glucose cotransporter SGLT1. Ddi1-like proteins play a significant role in cell cycle control, growth control, and trafficking in yeast and may play a crucial role in embryogenesis in higher eukaryotes.

Pssm-ID: 340494 **Cd Length:** 73 **Bit Score:** 42.54 **E-value:** 4.76e-07

```
                         10        20        30        40        50        60        70
                 ....*....|....*....|....*....|....*....|....*....|....*....|....*....|.
Query_22360  16  IKVHLADEALLLPLTVRPRDTLSDLRAqLVGQ--GVSSWKRAFYYNARRL-DDHQTVRDARLQDGSVLLLV 83
Cdd:cd01796   3  LTVTTEDDDRLFSLEVSPDMTLEDLKA-LCEAetGIPAAEQVLLHNGQPLtDDKKTLEALGLKDGDLLLLR 72
```

UBQ	smart00213	Ubiquitin homologues	28-83	7.61e-06

Ubiquitin homologues; Ubiquitin-mediated proteolysis is involved in the regulated turnover of proteins required for controlling cell cycle progression

Pssm-ID: 214563 **Cd Length:** 72 **Bit Score:** 39.55 **E-value:** 7.61e-06

```
                          10        20        30        40        50
                    ....*....|....*....|....*....|....*....|....*....|....*..
Query_22360      28 PLTVRPRDTLSDLRAQLVG-QGVSSWKRAFYYNARRLDDHQTVRDARLQDGSVLLLV 83
Cdd:smart00213   14 TLEVKPSDTVSELKEKIAElTGIPPEQQRLIYKGKVLEDDRTLADYGIQDGSTIHLV 70
```

ubiquitin	pfam00240	Ubiquitin family	28-83	1.47e-05

Ubiquitin family; This family contains a number of ubiquitin-like proteins: SUMO (smt3 homolog), Nedd8, Elongin B, Rub1, and Parkin. A number of them are thought to carry a distinctive five-residue motif termed the proteasome-interacting motif (PIM), which may have a biologically significant role in protein delivery to proteasomes and recruitment of proteasomes to transcription sites.

Pssm-ID: 333953 **Cd Length:** 71 **Bit Score:** 38.72 **E-value:** 1.47e-05

```
                         10        20        30        40        50
                   ....*....|....*....|....*....|....*....|....*....|....*..
Query_22360     28 PLTVRPRDTLSDLRAQL-VGQGVSSWKRAFYYNARRLDDHQTVRDARLQDGSVLLLV 83
Cdd:pfam00240   11 TLEVKPTDTVLQLKEKIaDREGVPPDQQRLIYKGKVLEDDQTLGEYGIEDGSTIHLV 67
```

Supplementary Figure S2. TINCR protein contains a ubiquitin-like fold. The amino acid sequence of human TINCR protein was used as a query. The search for conserved domains within a protein sequence was performed at the website https://www.ncbi.nlm.nih.gov/Structure/cdd/wrpsb.cgi? using default parameters (Search against database: CDD v3.17 - 52910 PSSMs; Expect value threshold: 0.01; Apply low-complexity filter: no; Composition based statistics adjustment: yes). The three top domain hits are shown. References: Marchler-Bauer A, Bryant SH. CD-Search: protein domain annotations on the fly. Nucleic Acids Res. 2004;32:327-331. Marchler-Bauer A et al. CDD: a Conserved Domain Database for the functional annotation of proteins. Nucleic Acids Res. 2011;39:225-9. Marchler-Bauer A et al. CDD: NCBI's conserved domain database. Nucleic Acids Res. 2015;43:222-6. Marchler-Bauer A et al. CDD/SPARCLE: functional classification of proteins via subfamily domain architectures. Nucleic Acids Res. 2017;45:200-3.
